# METTL3 promotes colorectal carcinoma progression by regulating the m6A–CRB3–Hippo axis

**DOI:** 10.1186/s13046-021-02227-8

**Published:** 2022-01-10

**Authors:** Jiashu Pan, Feng Liu, Xiaoli Xiao, Ruohui Xu, Liang Dai, Mingzhe Zhu, Hanchen Xu, Yangxian Xu, Aiguang Zhao, Wenjun Zhou, Yanqi Dang, Guang Ji

**Affiliations:** 1 China-Canada Center of Research for Digestive Diseases (ccCRDD), Institute of Digestive Diseases, Longhua Hospital, Shanghai University of Traditional Chinese Medicine, Shanghai, 200032 China; 2 Department of Digestive Disease, Longhua Hospital, Shanghai University of Traditional Chinese Medicine, Shanghai, 200032 China; 3 Department of Oncology, Longhua Hospital, Shanghai University of Traditional Chinese Medicine, Shanghai, 200032 China; 4 School of Public Health, Shanghai University of Traditional Chinese Medicine, Shanghai, 200032 China; 5 Department of General Surgery, Longhua Hospital, Shanghai University of Traditional Chinese Medicine, Shanghai, 200032 China

**Keywords:** Colorectal carcinoma, RNA N6-methyladnosine, Methyltransferase-like 3, Crumbs3, Hippo pathway

## Abstract

**Background:**

Colorectal carcinoma (CRC) is the third most common cancer and second most common cause of cancer-related deaths worldwide. Ribonucleic acid (RNA) N6-methyladnosine (m6A) and methyltransferase-like 3 (METTL3) play key roles in cancer progression. However, the roles of m6A and METTL3 in CRC progression require further clarification.

**Methods:**

Adenoma and CRC samples were examined to detect m6A and METTL3 levels, and tissue microarrays were performed to evaluate the association of m6A and METTL3 levels with the survival of patients with CRC. The biological functions of METTL3 were investigated through cell counting kit-8, wound healing, and transwell assays. M6A epitranscriptomic microarray, methylated RNA immunoprecipitation-qPCR, RNA stability, luciferase reporter, and RNA immunoprecipitation assays were performed to explore the mechanism of METTL3 in CRC progression.

**Results:**

M6A and METTL3 levels were substantially elevated in CRC tissues, and patients with CRC with a high m6A or METTL3 levels exhibited shorter overall survival. METTL3 knockdown substantially inhibited the proliferation, migration, and invasion of CRC cells. An m6A epitranscriptomic microarray revealed that the cell polarity regulator Crumbs3 (CRB3) was the downstream target of METTL3. METTL3 knockdown substantially reduced the m6A level of CRB3, and inhibited the degradation of CRB3 mRNA to increase CRB3 expression. Luciferase reporter assays also showed that the transcriptional level of wild-type CRB3 significantly increased after METTL3 knockdown but not its level of variation. Knockdown of YT521-B homology domain–containing family protein 2 (YTHDF2) substantially increased CRB3 expression. RNA immunoprecipitation assays also verified the direct interaction between the YTHDF2 and CRB3 mRNA, and this direct interaction was impaired after METTL3 inhibition. In addition, CRB3 knockdown significantly promoted the proliferation, migration, and invasion of CRC cells. Mechanistically, METTL3 knockdown activated the Hippo pathway and reduced nuclear localization of Yes1-associated transcriptional regulator, and the effects were reversed by CRB3 knockdown.

**Conclusions:**

M6A and METTL3 levels were substantially elevated in CRC tissues relative to normal tissues. Patients with CRC with high m6A or METTL3 levels exhibited shorter overall survival, and METTL3 promoted CRC progression. Mechanistically, METTL3 regulated the progression of CRC by regulating the m6A–CRB3–Hippo pathway.

**Supplementary Information:**

The online version contains supplementary material available at 10.1186/s13046-021-02227-8.

## Background

Colorectal carcinoma (CRC) is the third most common cancer and second most common cause of cancer-related deaths worldwide [1]. Because of tumor metastasis and other complications, the mortality rate of CRC remains high. Therefore, a timely clarification of the molecular mechanism and effective therapeutic targets of CRC is warranted.

Ribonucleic acid (RNA) N6-methyladnosine (m6A) is the most common and abundant RNA modification in eukaryotes [2, 3]. M6A modification is mainly mediated by m6A methyltransferases, demethylases, and reader proteins. M6A methyltransferases mainly comprise methyltransferase-like 3 (METTL3) [4], methyltransferase-like 14 (METTL14) [5], Wilms tumor 1–associated protein (WTAP) [6], RNA binding motif protein 15 [7], and vir-like m6A methyltransferase associated [8]. M6A demethylases mainly comprise fat-mass and obesity-associated protein (FTO) [9] and alkylation repair homolog protein 5 (ALKBH5) [10]. Reader proteins mainly comprise YT521-B homology domain–containing family protein 1/2/3 (YTHDF1/2/3), YT521-B homology domain-–containing 1/2, insulin-like growth factor 2 mRNA binding proteins 1/2/3, and heterogeneous nuclear ribonucleoprotein family [11–13]. Several studies have verified that m6A modification plays a key role in cancer progression [9, 14]. METTL3 and METTL14 can regulate the progression of multiple types of cancer, including bladder cancer [15, 16], gastric cancer [17, 18], cervical cancer [19], hepatocellular carcinoma [14, 20], and pancreatic cancer [21]. FTO plays a central role in oral squamous cell carcinoma [22], hepatocellular carcinoma [23], bladder cancer [24], and acute myeloid leukemia [9, 25]. Although studies have also demonstrated that m6A modification plays a key role in CRC [4, 12], the function and regulatory mechanism of m6A in CRC progression remain unclear.

In the present study, we verified the m6A and METTL3 levels in both adenomas (precancerous lesions of CRC) and CRC tissues, and clarified the function of METTL3 in CRC progression. Moreover, we identified Crumbs3 (CRB3) as a downstream target of METTL3 and verified the function of CRB3 in CRC. Then, we discovered that METTL3 and CRB3 regulate CRC progression through the Hippo pathway, and the function of METTL3 in CRC progression was rescued by CRB3. Therefore, our findings elucidated the role of METTL3, and we provided a potential new treatment strategy against CRC.

## Materials and methods

### Clinical tissue specimens

Thirty CRC, 30 adenoma, and 30 adjacent normal (normal) tissues were obtained during surgery from Longhua Hospital, which is affiliated with the Shanghai University of Traditional Chinese Medicine. The diagnosis of CRC and adenoma was confirmed according to pathological evidence. Tissues were snap-frozen in liquid nitrogen and stored at − 80 °C before detection was performed. This study was approved by the Ethics Committee of Longhua Hospital (2019LCSY020), and informed consent was obtained from all participants.

### RNA m6A quantification assay

M6A level was assayed using an RNA m6A quantification kit (ab185912, Abcam, USA) per the protocol used in a previous study [26]. Briefly, 200 ng of RNA was incubated for 60 min with capture antibody, after which detection antibody and enhancer solution were added. Finally, the samples were incubated with developer solution for 10 min. Absorbance was detected at a wavelength of 450 nm.

### Immunohistochemistry

Tissue samples from the normal, adenoma, and CRC groups were fixed and then cut into 4-μm sections for immunohistochemistry (IHC). Tissue microarrays (TMAs) were obtained from Shanghai Outdo Biotech Co., Ltd. (Shanghai, China), and IHC was performed. In brief, tissue samples were treated with EDTA, as an antigen retrieval solution, and then incubated with m6A antibodies (1:100, 56,593, CST, USA) and METTL3 antibodies (1:500, ab195352, Abcam, USA) overnight at 4 °C. Subsequently, secondary antibodies were incubated for 1 h at 37 °C. Finally, the samples were stained and imaged. The scores for staining intensity were determined according to a staining intensity scale that ranged from 0 to 3+ points (0 for no staining, 1+ for weak immunoreactivity, 2+ for moderate immunoreactivity, and 3+ for strong immunoreactivity). Positive percentage was scored as follows: 0 for negative cells, 1+ for 1–25%, 2+ for 26–50%, 3+ for 51–75%, and 4+ for 76–100%. The intensity and positive proportion scores were then multiplied to obtain a composite score, which recorded as the scores of IHC. The composite score ranged from 0 to 12; a below average score indicates low expression, whereas an above average score indicates high expression. The clinical characteristics of the samples are summarized in Additional file 4: Tables S2 and S4.

### Immunofluorescence

The m6A antibody (1:300, 56,593, CST), METTL3 antibody (1:1000, ab195352, Abcam), and CRB3 antibody (1:500, PA5–53092, Thermo Fisher Scientific, USA) were used. Samples were incubated with m6A, METTL3, and CRB3 antibodies overnight at 4 °C. Subsequently, secondary antibodies conjugated with Alexa Fluor were incubated for 1 h at 37 °C. Finally, the samples were stained and imaged.

### M6A methylase activity assay

Nuclear extracts of adenoma and CRC tissues were performed using a nuclear extraction kit I (OP-0002, Epigentek, USA). Briefly, tissues were cut into small pieces, and homogenized. Then they were incubated on ice for 15 min and centrifuged. Finally, the supernatant was collected. Then m6A methylase activity was performed using m6A methylase activity/inhibition assay kit (P-9019, Epigentek, USA) per the protocol. In brief, 80 μl of binding solution and 2 μl of m6A methylase substrate were added into each strip well, which were incubated at 37 °C for 90 min. Then binding solution was removed, and 46 μl of working methylase buffer and 4 μl of nuclear extracts were added into each well. Fifty microlitre of the diluted capture antibody were added and signal detection was performed at 450 nm. Finally, m6A methylase activity was calculated per the protocol.

### Cell culture and transfection

Normal colon cells (FHC) and CRC cells (HCT116, HT29, SW480, and SW620; Shanghai Cell Bank, Shanghai, China) were cultured in Dulbecco’s Modified Eagle Medium supplemented with 10% fetal bovine serum (FBS) and penicillin/streptomycin (100 U/mL) in an incubator with 5% CO_2_ at 37 °C. In addition, 293 T cells obtained from the American Type Culture Collection were cultured in Roswell Park Memorial Institute 1640 Medium with 10% FBS and penicillin/streptomycin (100 U/mL; Gibco, USA). METTL3 short hairpin RNA plasmid, CRB3 short hairpin RNA plasmid, or negative control (Genomeditech, China) were transfected using FuGene HD transfection reagent (E2311, Promega, USA) per the protocol used in a previous study [27].

### Cell counting kit-8 assay

After the transfection of HCT116 and SW620 cells was completed, these cells were seeded on 96-well plates at a concentration of 2 × 10^4^ cells and cultured for 0, 24, 48, and 72 h. Subsequently, 10-μL cell counting kit-8 (CCK8) was added to each well. After incubation at 37 °C for 1 h, the absorbance value was detected at 450 nm.

### Wound healing assay

After the transfection of HCT116 and SW620 cells was completed, these cells were seeded on a six-well dish with a culture insert (ibidi, Germany) at a concentration of 3 × 10^4^ cells. After 24 h, the culture insert was removed, and the cells were washed twice with phosphate buffer. Thereafter, 2-mL serum-free medium was added to each dish, which was then set aside for 48 h. Images were captured, and the wound area was measured using ImageJ software (National Institutes of Health, USA).

### Transwell assay

Six-well plates with 8-μm chambers (Corning, USA) were used to assess cellular migration (without Matrigel) or invasion (with Matrigel). In brief, transfected HCT116 and SW620 cells were seeded in six-well plates at a concentration of 1 × 10^5^ cells. In total, 200-μL serum-free medium was added to the upper chamber, and 600-μL of medium with 30% FBS was added to the lower chamber, and they were set aside for 48 h. The cells were then fixed with 4% paraformaldehyde for 30 min and stained with 0.1% crystal violet solution for 15 min. Five fields were randomly selected to calculate the area of migrating or invading cells.

### Quantitative real-time polymerase chain reaction

Total RNA was extracted using TRIzol reagent (Ambion, USA). Complementary DNA was synthesized using an EVM-MLV reverse transcription kit (Aikeri Biotech, China). The amplification reaction was performed using the SYBR-Green quantitative real-time polymerase chain reaction (qPCR) kit (Thermo Fisher Scientific, USA). Gene expression was normalized using β-actin. The primers are listed in Additional file 4: Table S1.

### Western blotting

Cells were collected and lysed, and protein concentration was determined. The protein was separated and transferred to a polyvinylidene difluoride membrane followed by incubation with 5% milk at room temperature for 1 h. The membrane was incubated at 4 °C overnight with the following antibodies: METTL3 (1:1000, 86,132, CST), CRB3 (1:1000, NBP1–98328, Novus Biologicals, USA), YTHDF2 (1:1000, 80,014, CST), macrophage stimulating 1 (MST1, 1:1000, 3682, CST), phospho-MST1 (1:1000, 49,332, CST), salvador family WW domain containing protein 1 (SAV1, 1:1000, 13,301, CST), large tumor suppressor kinase 1 (LATS1, 1:1000, 3477, CST), phospho-LATS1 (1:1000, 8654, CST), MOB kinase activator 1 (MOB1, 1:1000, 13,730, CST), phospho-MOB1 (1:1000, 8699, CST), Yes1-associated transcriptional regulator (YAP, 1:1000, 4912, CST), phospho-YAP (1:1000, 13,008, CST), histone (1:1000, 4499S, CST), and β-actin (1:1000, 4970 s, CST). The secondary antibody was then added, and the membrane was incubated at room temperature for 1 h; protein expression was observed using a chemiluminescence gel imaging system (Tanon 5200, China).

### Human m6A epitranscriptomic microarray analysis

Total RNA was quantified using the NanoDrop ND-1000. The sample preparation and microarray hybridization were performed per Arraystar’s standard protocols. In brief, the total RNAs were immunoprecipitated with anti-m6A antibody. The modified RNAs were eluted from the immunoprecipitated magnetic beads as the “IP.” The unmodified RNAs were recovered from the supernatant as “Sup.” The IP and Sup RNAs were labeled with Cy5 and Cy3, respectively, using the Arraystar Super RNA Labeling Kit. The RNAs were combined and hybridized onto an Arraystar Human m6A Epitranscriptomic Microarray (8x60K, Arraystar). After the slides were washed, the arrays were scanned using an Agilent Scanner G2505C.

### Data processing and analysis

Agilent Feature Extraction software was used to analyze acquired array images. The raw intensity levels of IP (Cy5-labeled) and Sup (Cy3-labeled) were normalized. After the completion of normalization, probe signals were retained for further m6A methylation level and m6A-quantity analysis. M6A methylation level was calculated by determining the percentage of modification according to the IP (Cy5-labeled) and Sup (Cy3-labeled) normalized intensity levels. M6A quantity was calculated by determining the m6A methylation amount according to the IP (Cy5-labeled) normalized intensity levels. The differentially m6A-methylated RNAs of the two comparison groups were identified by using filtering with the thresholds of > 1.5 for fold change and < 0.05 for *P* value.

### Methylated RNA immunoprecipitation-qPCR

The m6A level of CRB3 was determined using a methylated RNA immunoprecipitation-qPCR assay per the protocol used in previous studies [4, 14]. In brief, 100 μg of total RNA was purified using an mRNA purification kit (61,006, Invitrogen, USA) and subsequently sonicated. And 2 μg of the RNA was saved as the input. These RNA fragments were further immunoprecipitated with 5 μg of anti-m6A antibody (202,003, Synaptic Systems). Samples were incubated using protein A/G magnetic beads (88,803, Thermo Fisher Scientific, USA). The enriched RNA was analyzed through qPCR, and the m6A enrichment was normalized using input. The primers are listed in Additional file 4: Table S4.

### RNA stability assay

HCT116 cells were seeded on 6-well plates for 24 h, and then treated with 5 μg/mL actinomycin D (MCE, USA) at the 0, 2, 4, 8, 24 h. Total RNA was then isolated by using TRIzol (Ambion) and analyzed through qPCR. The mRNA expression for each group at a given time was calculated and normalized using β-actin.

### RNA immunoprecipitation assay

RNA immunoprecipitation (RIP) assay was performed using the Magna RIP RNA-Binding Protein Immunoprecipitation Kit (17–700, Millipore, USA) per the manufacturer’s instructions. In brief, the cell samples were collected and incubated using magnetic beads with 5 μg of mouse immunoglobulin G (17–700, Millipore) or YTHDF2 (80,014, CST). Then protein-RNA complexes were then precipitated with a magnetic separator, and the supernatant was discarded. Thereafter, each immunoprecipitate was incubated with proteinase K, and the RNA was purified. Finally, the relative interaction between YTHDF2 and CRB3 transcripts was determined through qPCR and normalized to the input.

### Double luciferase reporter assay

To perform this assay, 293 T cells were seeded on 24-well plates and transfected using FuGene HD transfection reagent (E2311, Promega) per the protocol used in a previous study [28]. In brief, 293 T cells were transfected with CRB3 wild type (CRB3-WT; Genomeditech) or CRB3 variant (CRB3-Mut; Genomeditech) plasmid with or without METTL3 knockdown for 6 h. Luciferase activity was detected using the dual-luciferase reporter system (E1910, Promega).

### Statistical analysis

Statistical analysis was conducted using SPSS 24.0. Data were assessed using a two-tailed Student’s t test. Survival curves were generated using the Kaplan–Meier method and compared using the log-rank test. Survival data were analyzed through univariate and multivariate Cox regression analyses. The distribution differences of the variables were analyzed using Pearson’s chi-square test. A *P* value of < 0.05 was regarded as statistically significant.

## Results

### M6A levels were substantially elevated in both adenoma and CRC and were associated with poor prognosis

**Fig. 1:**
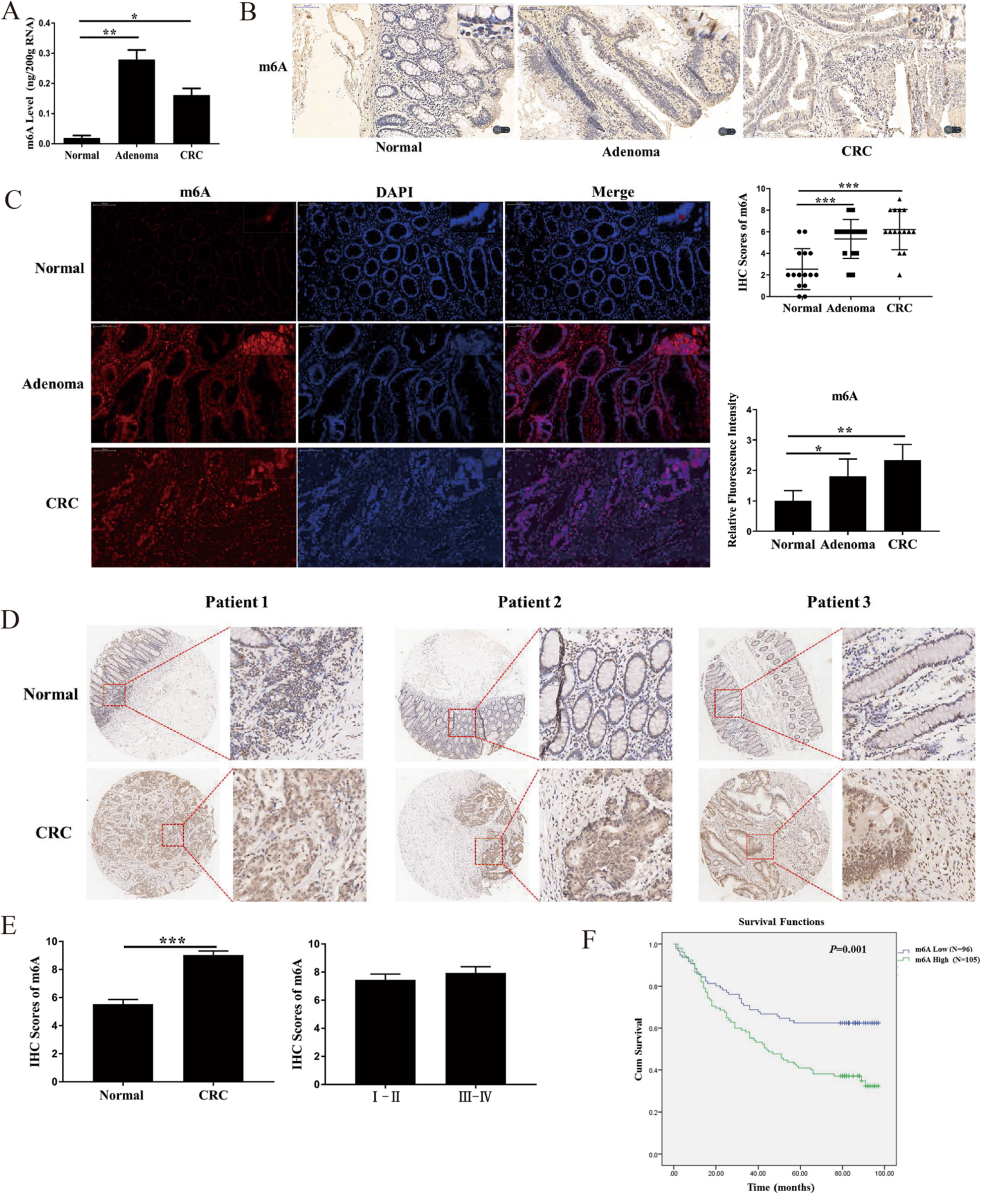
N6-methyladnosine level was substantially increased in both adenoma and colorectal carcinoma and associated with poor prognosis. N6-methyladnosine (m6A) levels in both adenoma and colorectal carcinoma (CRC) were assayed through (**a**) Methylation quantification assay, **b** Immunohistochemistry (IHC), and (**c**) Immunofluorescence. **d** Representative images of IHC staining for m6A level as obtained from 201 CRC tissues and 159 normal tissues; **e** IHC staining scores of m6A; **f** Kaplan–Meier overall survival analysis of m6A expression in patients with CRC. Data are presented as means ± standard deviations (SDs). ^*^P < 0.05, ^**^P < 0.01, ^***^P < 0.001

A study indicated that approximately 85% of CRCs may have transformed from adenomas [29]; therefore, we examined the m6A levels in both adenoma and CRC tissues. The results revealed substantially higher m6A levels in both adenoma and CRC groups relative to the normal group (Fig. 1a–c). A TMA was performed to explore the correlation between m6A and survival in patients with CRC. The results indicated a substantially higher m6A level in the CRC group relative to the normal group (Fig. 1d and e, Additional file 4: Table S2). Furthermore, patients with CRC with high m6A level exhibited shorter overall survival (Fig. 1f, Additional file 4: Table S3).

### METTL3 level was substantially elevated in CRC and was associated with poor prognosis

**Fig. 2:**
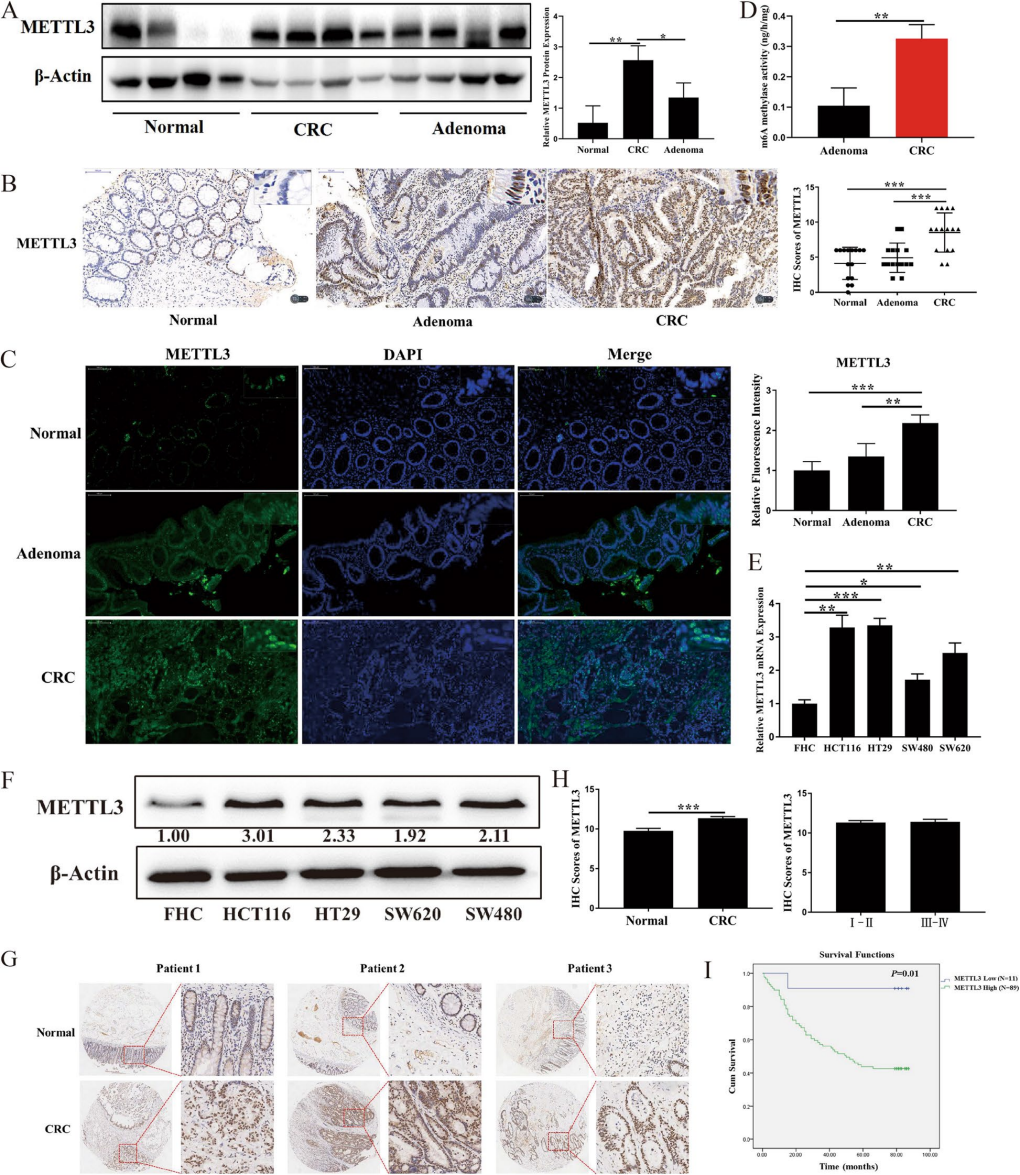
Methyltransferase-like 3 was substantially increased in colorectal carcinoma and associated with poor prognosis. Methyltransferase-like 3 (METTL3) expression in both adenoma and colorectal carcinoma (CRC) was assayed through (**a**) Western blot, **b** Immunohistochemistry (IHC), and **c** Immunofluorescence. **d** N6-methyladnosine (m6A) methylase activity was assayed. **e** METTL3 mRNA level in CRC cells; **f** METTL3 protein level in CRC cells; **g** Representative images of IHC staining for METTL3 level as obtained from 100 CRC tissues and 80 normal tissues; **h** METTL3 IHC staining scores; **i** Kaplan–Meier overall survival analysis of METTL3 expression in patients with CRC. Data are presented as means ± standard deviations (SDs). ^*^P < 0.05, ^**^P < 0.01, ^***^P < 0.001

M6A modification is regulated by RNA methyltransferase and demethylase, hence we assessed the expression of methyltransferase (METTL3, METTL14 and WTAP) and demethylase (FTO and ALKBH5) in both adenoma and CRC. The results indicated that the protein level of METTL3 was significantly higher in the CRC group relative to the normal and adenoma groups (Fig. 2a–c); this finding is consistent with the results in the database (Additional file 1: Fig. S1a). And mRNA level of METTL14 was significantly higher in the adenoma group but not in the CRC group (Additional file 1: Fig. S1c). Relative to the normal group, FTO was significantly lower in the adenoma group but not in the CRC group (Additional file 1: Fig. S1d). WTAP and ALKBH5 levels did not differ significantly among the three groups (Additional file 1: Fig. S1e and 1f). M6A methylase activity was significantly higher in the CRC group relative to the adenoma group (Fig. 2d). In CRC cells, the mRNA and protein levels of METTL3 were also substantially higher than in normal colon cells (Fig. 2e and f). To explore the correlation between METTL3 and survival of patients with CRC, a TMA was performed. The results indicated that the level of METTL3 was substantially higher in the CRC group relative to the normal group (Fig. 2g and h, Additional file 4: Table S4). The patients with CRC with high METTL3 level also exhibited shorter overall survival relative to those without a high METTL3 level (Fig. 2i, Additional file 4: Table S5), which suggests that METTL3 level can serve as a prognostic marker of CRC.

### METTL3 drove CRC proliferation and invasion

**Fig. 3:**
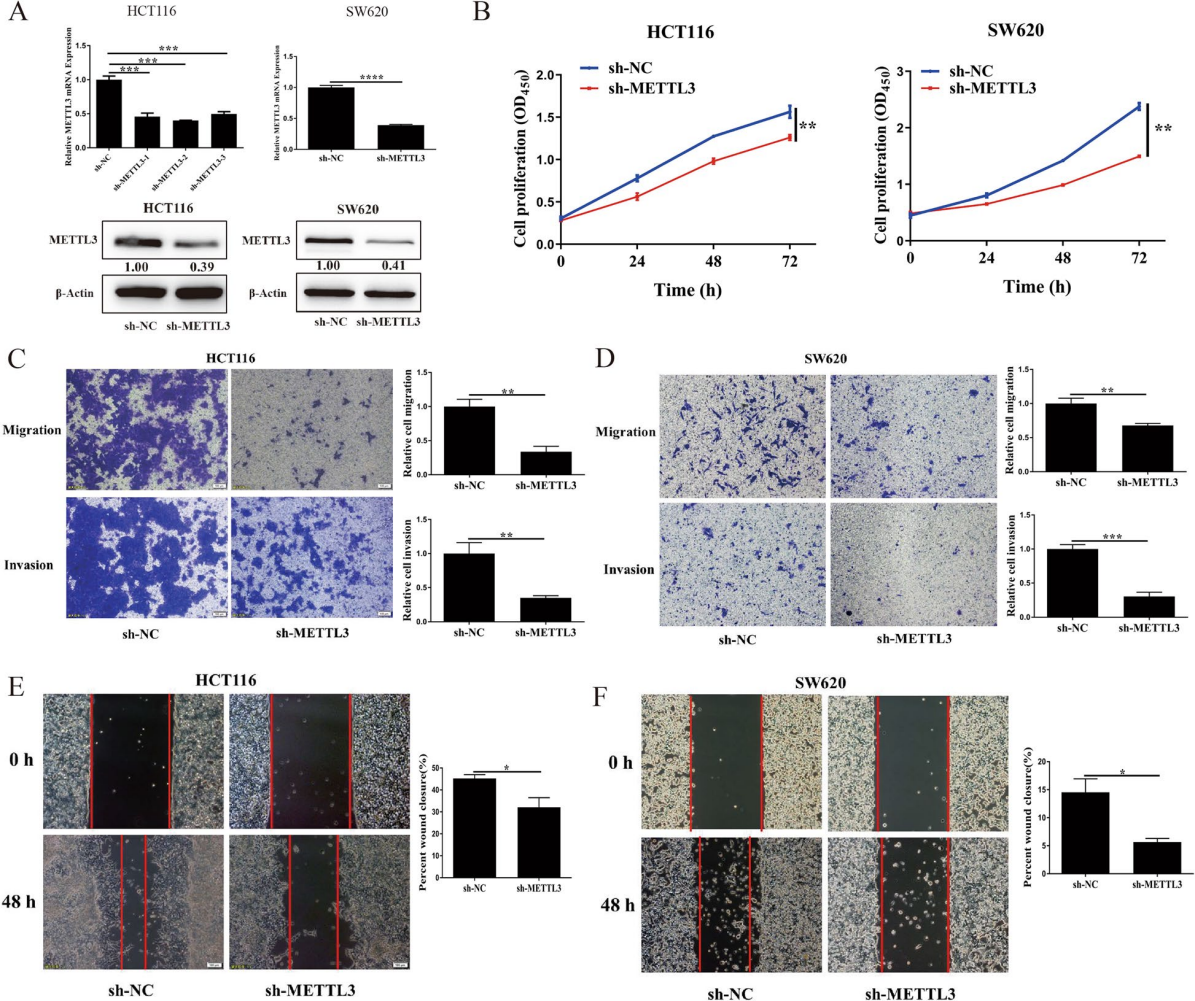
Methyltransferase-like 3 drives colorectal carcinoma proliferation and invasion. **a** Expression of Methyltransferase-like 3 (METTL3) in HCT116 and SW620 cells with METTL3 knockdown; **b** Proliferation of HCT116 and SW620 cells after METTL3 knockdown. Transwell assays with METTL3 knockdown for (**c**) HCT116 cells and (**d**) SW620 cells. Wound healing assay with METTL3 knockdown for (**e**) HCT116 cells and (**f**) SW620 cells. Data are presented as means ± standard deviations (SDs). ^*^P < 0.05, ^**^P < 0.01, ^***^P < 0.001

To investigate the function of METTL3 in CRC, METTL3 knockdown was performed in HCT116 and SW620 cells (Fig. 3a), and it significantly inhibited the proliferation of HCT116 and SW620 cells (Fig. 3b). Transwell assays indicated that METTL3 knockdown substantially inhibited the migration and invasion of HCT116 and SW620 cells (Fig. 3c and d), and METTL3 knockdown also significantly reduced the migration speed of HCT116 and SW620 cells (Fig. 3e and f).

### CRB3 was regulated by METTL3-mediated m6A modification

**Fig. 4:**
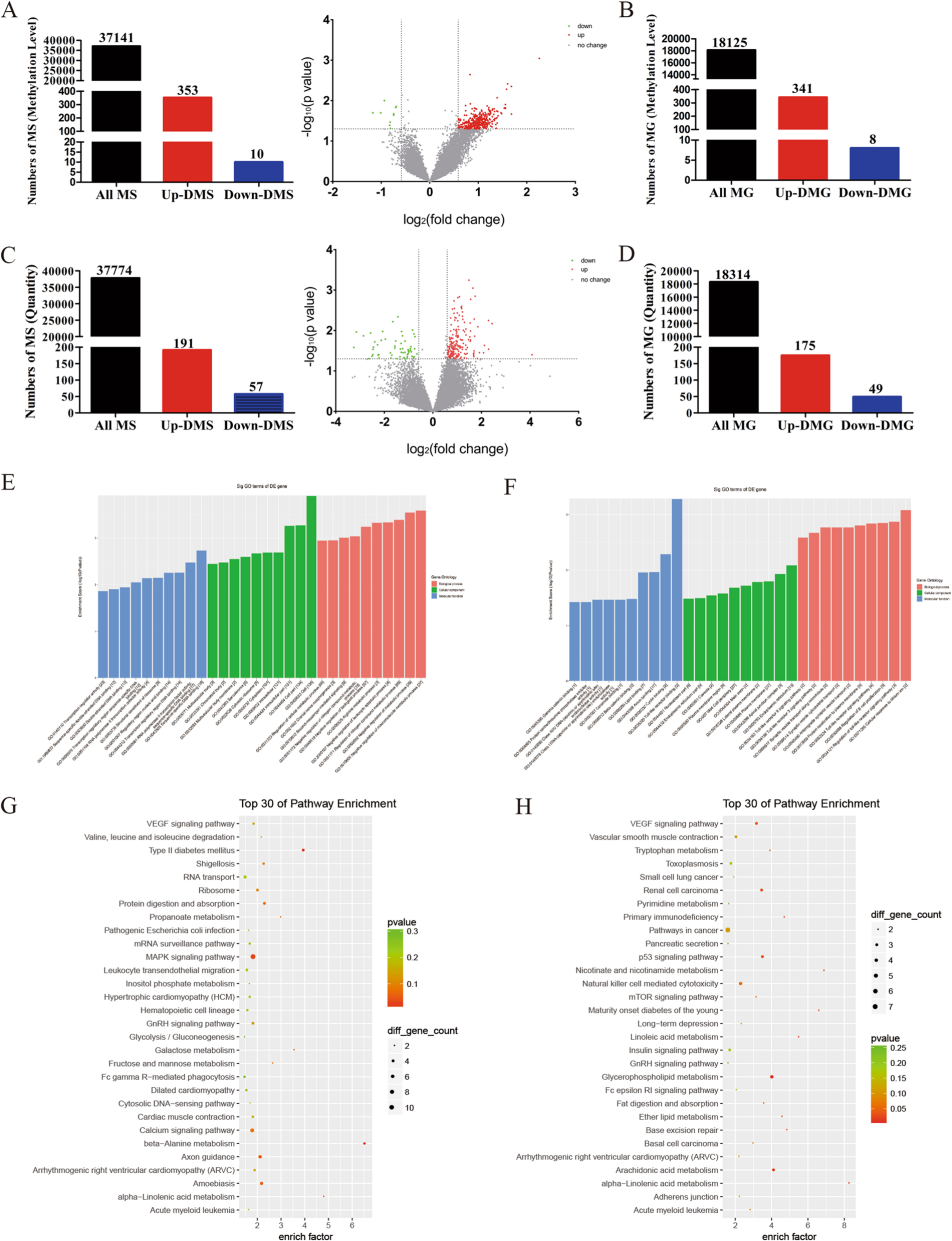
Analysis of epitranscriptomic microarray. Analysis of differentially N6-methyladnosine (m6A)-methylated sites (DMS) (**a**) and differentially m6A-methylated genes (DMG) (**b**) at m6A methylation level; Analysis of DMSs (**c**) and DMGs (**d**) at m6A quantity level; **e, f** Analysis of Gene Ontology at m6A methylation level and m6A quantity level; **g, h** Analysis of Kyoto Encyclopedia of Genes and Genomes at m6A methylation level and m6A quantity level

To investigate the potential mechanism of METTL3 in CRC progression, an epitranscriptomic microarray was performed, and the data obtained were analyzed. For m6A methylation, 363 differentially m6A-methylated sites (DMSs) were identified; they comprised 353 upregulated DMSs and 10 downregulated DMSs (Fig. 4a, Additional file 5). A further analysis revealed that these 363 DMSs belonged to 349 differentially m6A-methylated genes (DMGs), which comprised 341 upregulated DMGs and 8 downregulated DMGs (Fig. 4b). For m6A quantity, 248 DMSs were identified; they comprised 191 upregulated DMSs and 57 downregulated DMSs (Fig. 4c, Additional file 6). A further analysis revealed that these 248 DMSs belonged to 224 DMGs, which comprised 175 upregulated DMGs and 49 downregulated DMGs (Fig. 4d). Subsequently, main functions were identified using the Gene Ontology and Kyoto Encyclopedia of Genes and Genomes, which contain sequence-specific DNA binding, RNA polymerase II transcription factor activity, Toll-like receptor binding, beta-alanine metabolism, alpha-linolenic acid metabolism, and nicotinate and nicotinamide metabolism (Fig. 4e-h).

**Fig. 5:**
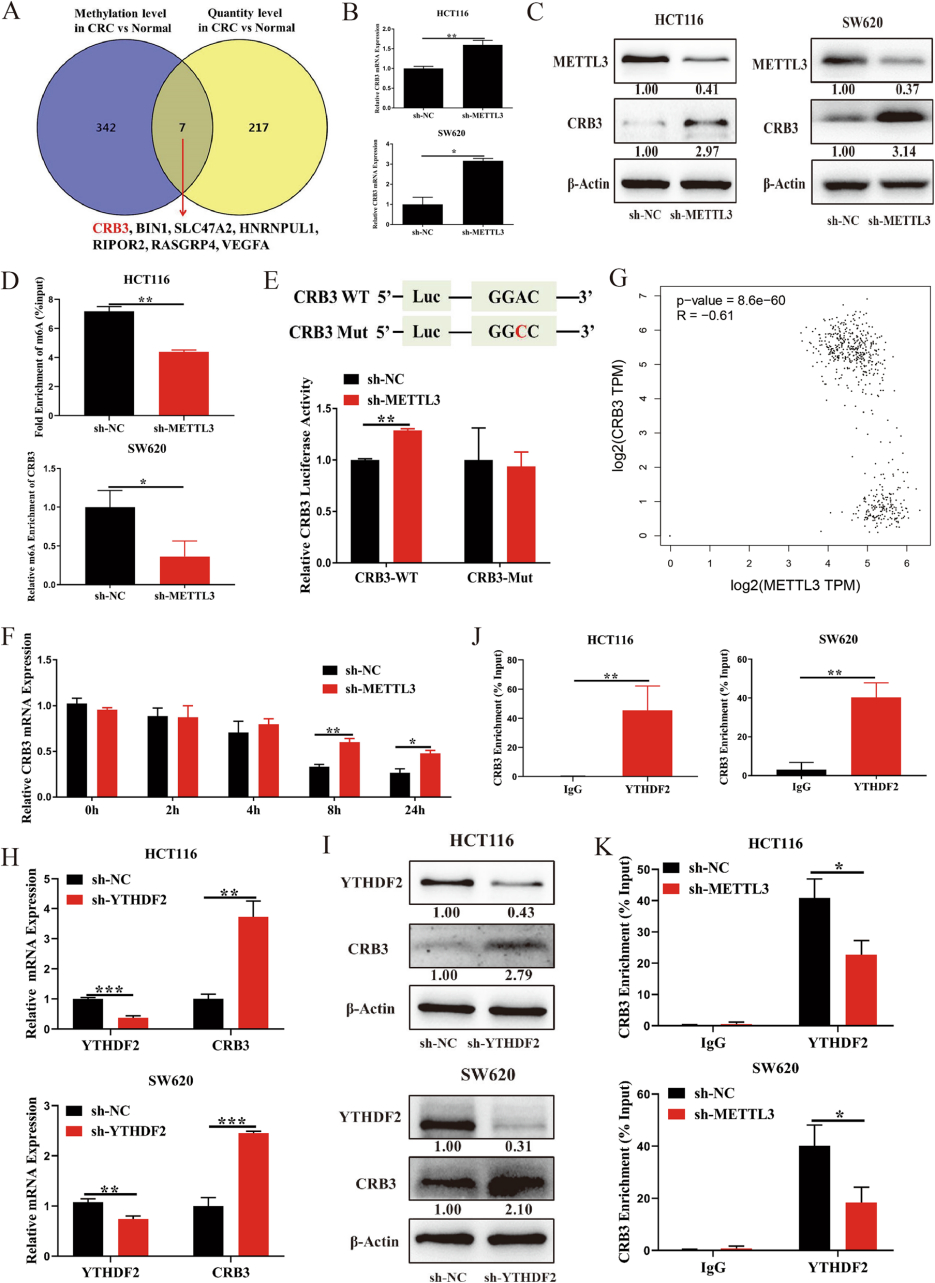
Methyltransferase-like 3 regulated the expression of crumbs3 in an N6-methyladnosine -dependent manner. **a** Overlapping differentially N6-methyladnosine (m6A)-methylated genes between m6A methylation level and quantity level as filtered through a Venn diagram; **b** Crumbs3 (CRB3) mRNA level as measured after methyltransferase-like 3 (METTL3) knockdown; **c** CRB3 protein level as measured after METTL3 knockdown; **d** The m6A level of CRB3 as measured after METTL3 knockdown; **e** Luciferase reporters performed to determine the effect of m6A modification on CRB3 expression; **f** CRB3 mRNA expression as detected with or without treatment of actinomycin D at indicated time points; **g** The correlation between METTL3 and CRB3 expression in The Cancer Genome Atlas database for CRC; **h** CRB3 mRNA level as measured after YTH domain–containing family protein 2 (YTHDF2) knockdown; **i** CRB3 protein level as measured after YTHDF2 knockdown; **j-k** The direct interaction was verified between the YTHDF2 and CRB3 mRNA. Data are presented as means ± standard deviations (SDs). ^*^P < 0.05, ^**^P < 0.01, ^***^P < 0.001

Seven overlapping DMGs between m6A methylation and quantity were filtered using a Venn diagram (Fig. 5a), and a qPCR analysis indicated a substantial increase in CRB3 level after METTL3 knockdown (Fig. 5b and c). The results obtained from The Cancer Genome Atlas (TCGA) database revealed a substantial downregulation of CRB3 level in the CRC group (Additional file 2: Fig. S2b). A survival analysis indicated that the patients with CRC with high CRB3 level exhibited higher overall survival and disease-free survival relative to those without high CRB3 level (Additional file 2: Fig. S2c). Although METTL3 knockdown also promoted bridging integrator 1 (BIN1) expression, a substantial increase in BIN1 expression was observed in the CRC group (Additional file 2: Fig. S2a and 2d). The survival analysis did not indicate a correlation between BIN1 level and survival in patients with CRC (Additional file 2: Fig. S2e). In addition, the m6A level of CRB3 was substantially reduced after METTL3 knockdown (Fig. 5d). Luciferase reporters were revealed to determine the effect of m6A modification on CRB3 expression. For the variant form of CRB3, the adenosine bases in m6A consensus sequences (GGAC) were replaced by cytosine; thus, m6A modification was abolished. The results indicated that the transcriptional level of wild-type CRB3 significantly increased after METTL3 knockdown but not its level of variation (Fig. 5e). An RNA stability assay revealed that METTL3 knockdown substantially inhibited the degradation of CRB3 mRNA (Fig. 5f). Moreover, data from the TCGA database indicated that METTL3 mRNA expression in CRC tissues was negatively associated with CRB3 levels (Fig. 5g). Studies have reported that YTHDF2 can target mRNAs by recognizing m6A motif in CRC [5, 12]; thus, we explored the effect of YTHDF2 on CRB3. The results indicated that the expression of YTHDF2 was also significantly higher in adenoma and CRC tissues relative to normal tissues (Additional file 3: Fig. S3), and YTHDF2 knockdown substantially increased the level of CRB3 (Fig. 5h and i). RIP assays also verified the direct interaction between the YTHDF2 and CRB3 mRNA, and this direct interaction was impaired after METTL3 inhibition in SW620 and HCT116 cells (Fig. 5j and k). The results indicated that METTL3 regulated the expression of CRB3 in an m6A-YTHDF2-dependent manner.

### CRB3 inhibited CRC proliferation and invasion

**Fig. 6:**
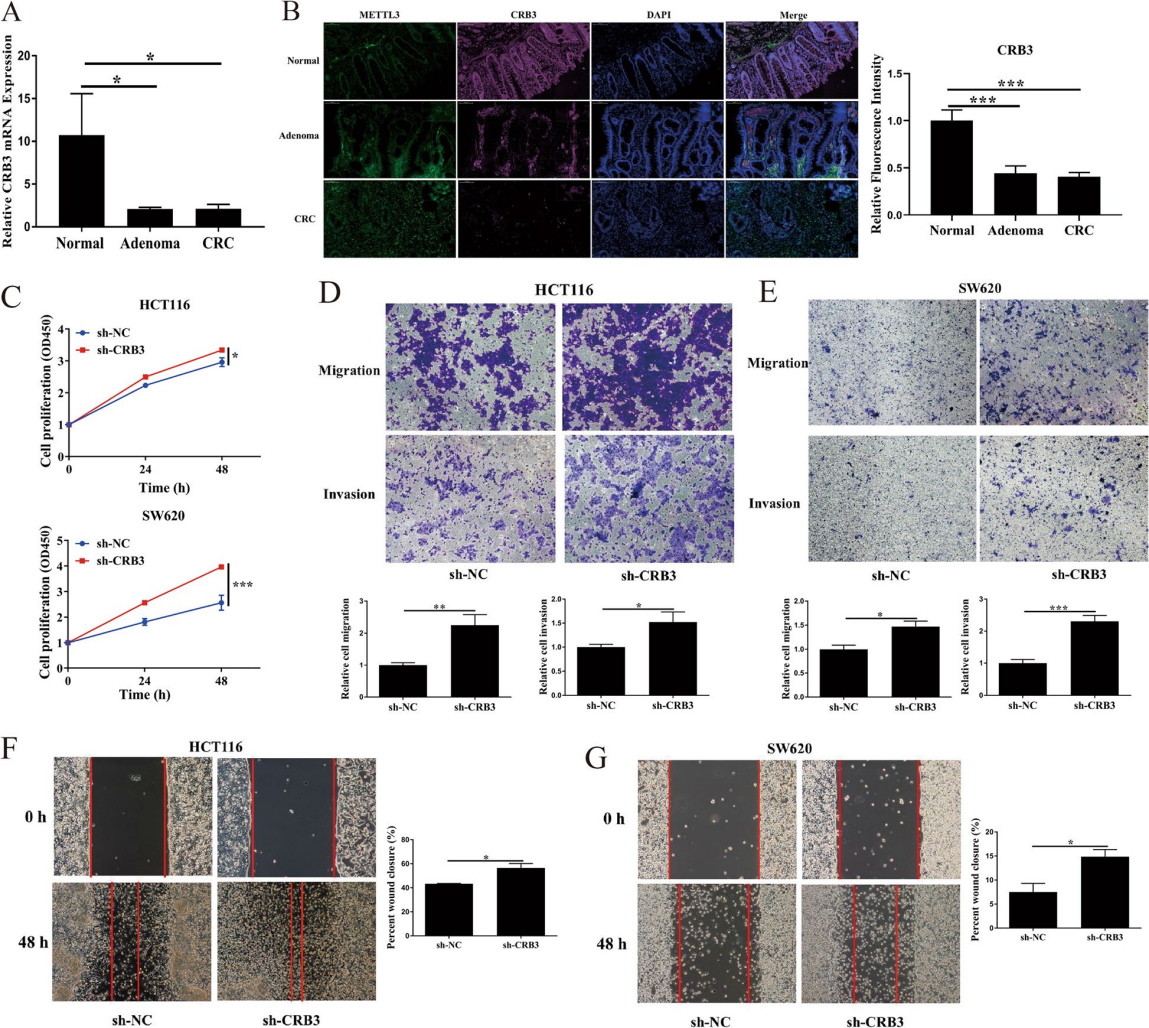
Crumbs3 inhibited colorectal carcinoma proliferation and invasion. Crumbs3 (CRB3) expression in both adenoma and colorectal carcinoma (CRC) as assayed through (**a**) qPCR and (**b**) Immunofluorescence; **c** Proliferation of HCT116 and SW620 cells as measured after CRB3 knockdown; Transwell assays performed with CRB3 knockdown in (**d**) HCT116 cells and (**e**) SW620 cells; Wound healing assay performed with CRB3 knockdown in (**f**) HCT116 cells and (**g**) SW620 cells. Data are presented as means ± standard deviations (SD). ^*^P < 0.05, ^**^P < 0.01, ^***^P < 0.001

Our results also indicated that CRB3 level was substantially lower in the adenoma and CRC groups than in the normal group, which was consistent with the data from TCGA database (Fig. 6a and b, Additional file 2: Fig. S2b). To further investigate the function of CRB3 in CRC, CRB3 knockdown was performed in HCT116 and SW620 cells. CRB3 knockdown significantly promoted the proliferation, migration and invasion of HCT116 and SW620 cells (Fig. 6c-e). CRB3 knockdown also significantly increased the migration speed of HCT116 and SW620 cells (Fig. 6f and g). These results verified that CRB3 regulated CRC progression.

### METTL3 regulated CRC proliferation and invasion by CRB3-hippo pathway

**Fig. 7:**
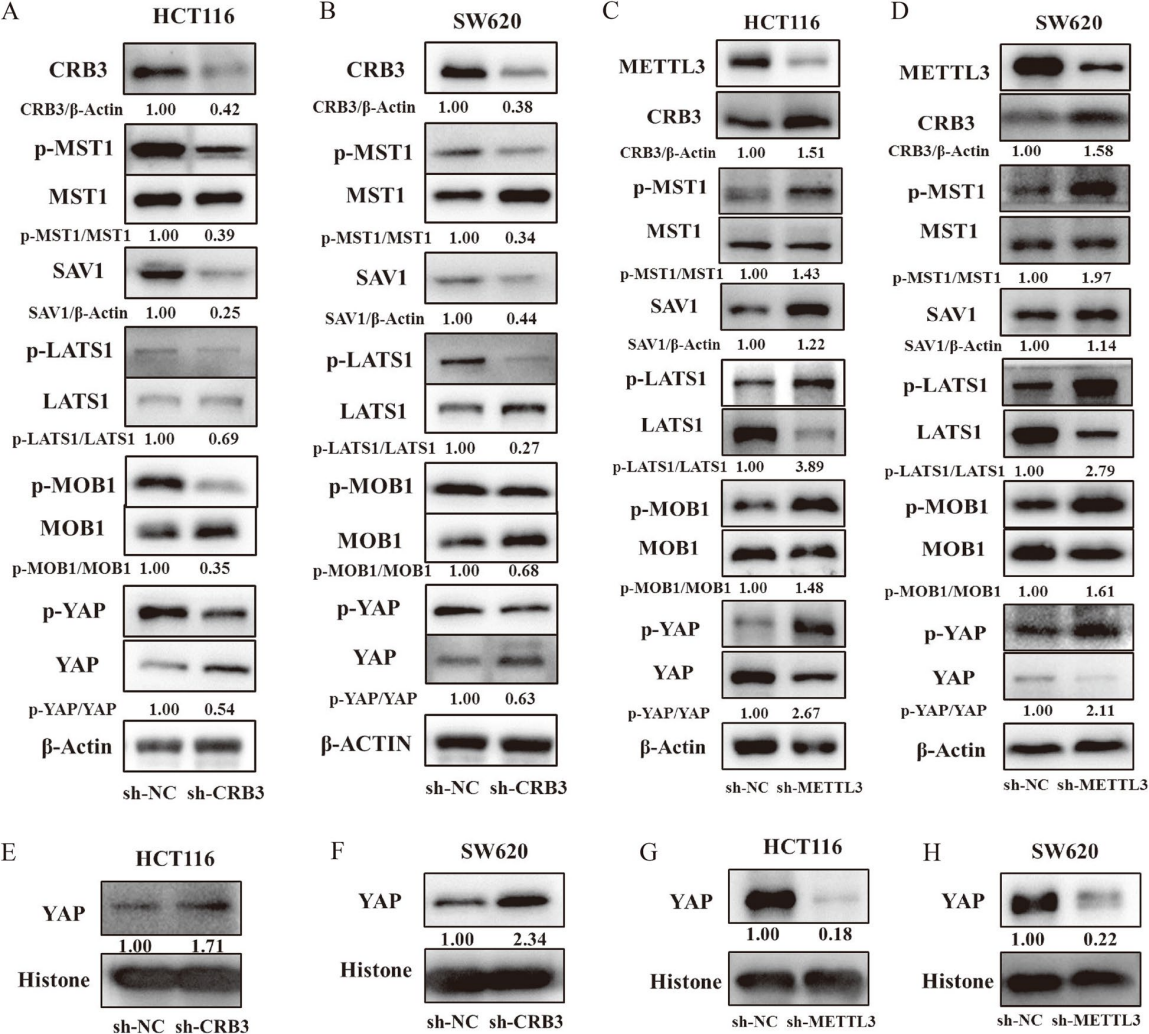
Methyltransferase-like 3 and crumbs3 could both regulate Hippo pathway. Hippo pathway as detected after (**a, b**) Crumbs3 (CRB3) knockdown or (**c, d**) Methyltransferase-like 3 (METTL3) knockdown in HCT116 and SW620 cells; Yes1-associated transcriptional regulator (YAP) protein level as detected after (**e, f**) CRB3 knockdown or (**g, h**) METTL3 knockdown in nuclei of HCT116 and SW620 cells. Data are presented as means ± standard deviations (SD). ^*^P < 0.05, ^**^P < 0.01, ^***^P < 0.001

Studies have demonstrated that CRB3 can regulate the Hippo pathway [30, 31]. In the present study, we discovered that CRB3 knockdown reduced MST1, LATS1, MOB1, and YAP phosphorylation levels, and it reduced the SAV1 levels in HCT116 and SW620 cells (Fig. 7a and b). Conversely, METTL3 knockdown substantially increased MST1, LATS1, MOB1, and YAP phosphorylation levels in HCT116 and SW620 cells (Fig. 7c and d). Studies have demonstrated that YAP enters the nucleus and acts as an oncogene; thus, we detected the level of YAP in nuclei. The results indicated that CRB3 knockdown markedly increased YAP protein levels in the nuclei of HCT116 and SW620 cells (Fig. 7e and f); however, METTL3 knockdown substantially reduced the YAP protein levels in the nuclei of HCT116 and SW620 cells (Fig. 7g and h).

**Fig. 8:**
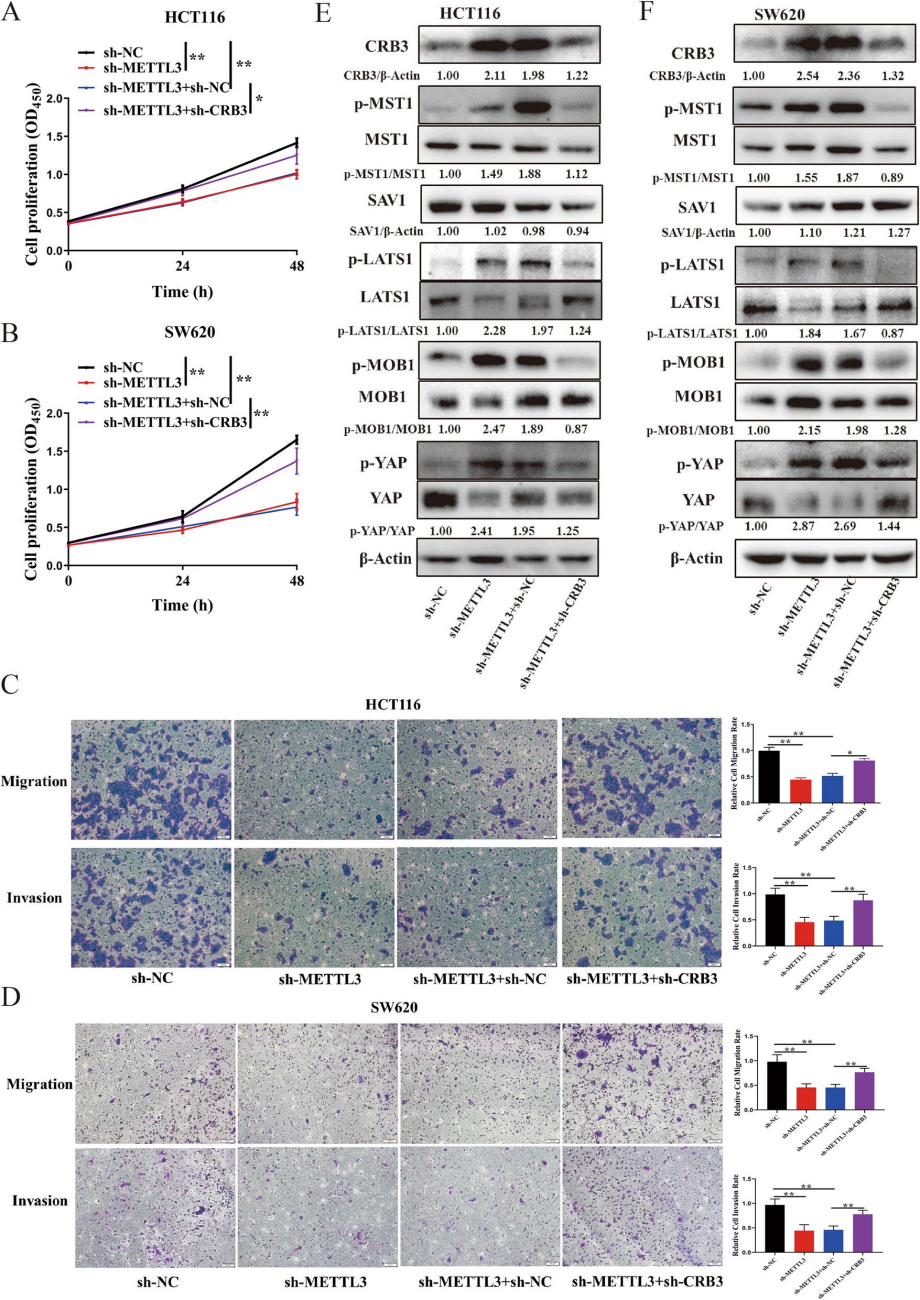
The effect of methyltransferase-like 3 on colorectal carcinoma progression was rescued by crumbs3. The proliferation of HCT116 (**a**) and SW620 cells (**b**) was rescued after crumbs3 (CRB3) knockdown; Migration and invasion was rescued after CRB3 knockdown in HCT116 (**c**) and SW620 cells (**d**); The activation of hippo pathway was reversed after CRB3 knockdown in HCT116 (**e**) and SW620 cells (**f**). Data are presented as means ± standard deviations (SD). ^*^P < 0.05, ^**^P < 0.01, ^***^P < 0.001

To test whether CRB3 functions downstream of METTL3, we knocked down CRB3 in the METTL3 knockdown background. The results revealed that the effects of METTL3 knockdown on cell proliferation, migration, and invasion were rescued by the CRB3 knockdown (Fig. 8a-d). In addition, CRB3 knockdown reduced the MST1, LATS1, MOB1, and YAP phosphorylation levels caused by METTL3 knockdown, indicating that CRB3 knockdown repressed activation of hippo pathway that was caused by METTL3 knockdown (Fig. 8e and f).

## Discussion

The incidence of CRC, which is a malignant tumor, has increased. The 5-year survival rate of patients with CRC is 65% [32], but it is extremely low in advanced-stage CRC. Therefore, techniques that aid the implementation of treatment strategies for CRC are urgently required because they can improve patient survival [27]. Studies have reported that m6A modification plays a key role in CRC [4, 5, 12, 33]. However, the changes to m6A in both adenoma and CRC are still unknown. In addition, the relationship between m6A level and the survival of patients with CRC requires further clarification. In the present study, we discovered that m6A levels are significantly increased in both adenoma and CRC tissues, indicating that m6A modification may be involved in the adenoma-to-CRC transition. A further examination indicated that the patients with CRC with high m6A level exhibited shorter overall survival.

M6A modification is mainly mediated by m6A methyltransferases, demethylases and reader proteins, and it regulates pre-mRNA splicing, miRNA processing, translation, and mRNA decay [34]. In the present study, we discovered that the protein level of METTL3 and m6A methylase activity were both significantly increased in CRC, indicating that METTL3 could be involved in the CRC progression. Furthermore, patients with CRC with high METTL3 level exhibited shorter overall survival, suggesting that METTL3 can also serve as a prognostic marker of CRC. These results are consistent with those reported by other studies [4, 35]. Subsequently, we verified the function of METTL3 in CRC. METTL3 knockdown inhibited the proliferation of HCT116 and SW620 cells, and it also substantially inhibited the migration and invasion of HCT116 and SW620 cells. These results indicated that METTL3 acts as an oncogene that promotes the progression of CRC. Studies have also reported on the key role of METTL3 in various cancers. METTL3 can regulate MALAT1 stabilization through m6A modification, and it activates NF-κB activity to promote the malignant progression of glioma [36]. METTL3 increases miR-1246 levels through m6A modification, thereby promoting non-small-cell lung cancer progression [37]. Moreover, METTL3 regulates the m6A modification of SPHK2 to promote the progression of gastric cancer [38]. These findings verified the role of METTL3 in cancers such as CRC. Therefore, METTL3 can be a new treatment target for cancers.

Through an m6A epitranscriptomic microarray analysis, we revealed that CRB3 might be the downstream target of METTL3. METTL3 knockdown substantially reduced the m6A level of CRB3 and inhibited the degradation of CRB3 mRNA, finally to increase CRB3 expression. Studies have reported that the m6A consensus sequences are GGAC [5]. Our study revealed that METTL3 knockdown increased the transcriptional level of CRB3. When the adenosine bases of GGAC in CRB3 were replaced by cytosine, the transcriptional level of CRB3 did not change. In addition, YTHDF2 could also regulate the CRC progression in an m6A-dependent manner [5, 12, 39]. In the present study, we discovered that YTHDF2 knockdown substantially increased the level of CRB3. The direct interaction between the YTHDF2 and CRB3 mRNA was also verified, and this direct interaction was impaired after METTL3 inhibition. These results indicated that METTL3 regulated the expression of CRB3 in an m6A-YTHDF2-dependent manner. CRB3 is a protein of cell polarity, and it is associated with contact inhibition [40]. Studies have demonstrated that CRB3 plays a central role in cancers such as CRC [30, 41, 42]. In the present study, we discovered that CRB3 levels in both adenoma and CRC were substantially lower than in normal tissues, and we also revealed that patients with CRC with high CRB3 level exhibited higher overall survival and disease-free survival. CRB3 knockdown significantly promoted the proliferation, migration, and invasion of HCT116 and SW620 cells. These results indicated that CRB3 regulates CRC progression.

The depletion of CRB3 can inhibit the Hippo pathway and lead to increased nuclear localization of YAP [30, 31, 43]. The Hippo pathway plays a crucial role in regulating CRC progression [44–47]. In the present study, we also observed that CRB3 knockdown inhibited the Hippo pathway and increased the nuclear localization of YAP, suggesting that CRB3 regulates CRC progression through the Hippo pathway. Conversely, METTL3 knockdown activated the Hippo pathway and reduced the nuclear localization of YAP. Finally, our results revealed that the effects of METTL3 knockdown on cell proliferation, migration, and invasion were rescued by the CRB3 knockdown. CRB3 knockdown reversed the activation of hippo pathway caused by METTL3 knockdown. Therefore, our study indicated that METTL3 facilitated CRC progression by regulating the m6A-CRB3-Hippo pathway, which is a novel mechanism for regulating CRC. Even though we demonstrated the regulatory mechanism of METTL3 in CRC, further studies are required. First, although a study reported that the selective first-in-class catalytic inhibitor of METTL3 (i.e., STM2457) can be used in treatment strategies for acute myeloid leukemia [48], the inhibitor of METTL3 has not yet been identified for the treatment of CRC. Therefore, further studies are required to identify the inhibitor of METTL3. Second, we discovered substantially elevated m6A levels in both adenoma and CRC; METTL3 level was substantially elevated in only CRC, not in adenoma. This suggests that other enzymes may also be involved in the m6A modification in adenoma, and further clarification was required.

## Conclusion

In summary, we demonstrated that m6A and METTL3 levels are significantly increased in CRC. Patients with CRC with high m6A or METTL3 levels exhibit shorter overall survival, and METTL3 promoted CRC progression. Mechanistically, METTL3 regulates the progression of CRC by regulating the m6A–CRB3–Hippo pathway. These findings provide a new perspective for the implementation of targeted therapy for CRC.

## Supplementary Information

**Additional file 1: Figure S1.** Expression of N6-methyladnosine methyltransferases and demethylases in both adenoma and colorectal carcinoma.

**Additional file 2: Figure S2.** Validation of downstream targets after methyltransferase-like 3 knockdown.

**Additional file 3: Figure S3.** The expression of YTH domain–containing family protein 2 was verified in adenoma and colorectal carcinoma tissues.

**Additional file 4: Table S1.** Primer sequences of mRNAs in real-time PCR experiments. **Table S2.** Correlation between N6-methyladnosine (m6A) level and multiple clinical characteristics. **Table S3.** Cox regression analyses of m6A level in patients with CRC. **Table S4.** Correlation between methyltransferase-like 3 (METTL3) expression and multiple clinical characteristics. **Table S5.** Cox regression analyses of METTL3 expression in patients with CRC. **Table S6.** Primer sequences of MeRIP-qPCR and RIP.

**Additional file 5.** mRNA methylation level in colorectal carcinoma vs. normal tissue.

**Additional file 6.** mRNA quantity level in colorectal carcinoma vs. normal tissue.

**Additional file 7: Figure S4.** All images of m6A immunofluorescence were showed.

## Data Availability

The datasets used and/or analyzed during the current study are available from the corresponding author on reasonable request.

## Abbreviations

CRC: Colorectal carcinoma
RNA: Ribonucleic acid
m6A: N6-methyladnosine
METTL3: Methyltransferase-like 3
CRB3: Crumbs3
METTL14: Methyltransferase-like 14
WTAP: Wilms tumor 1–associated protein
FTO: Fat-mass and obesity-associated protein
ALKBH5: Alkylation repair homolog protein 5
YTHDF1/2/3: YTH domain–containing family protein 1/2/3
Normal: Adjacent normal tissues
IHC: Immunohistochemistry
TMA: Tissue microarrays
FBS: Fetal bovine serum
CCK8: Cell counting kit-8
qPCR: Quantitative real-time polymerase chain reaction
MST1: Macrophage stimulating 1
SAV1: Salvador family WW domain containing protein 1
LATS1: Large tumor suppressor kinase 1
MOB1: MOB kinase activator 1
YAP: Yes1-associated transcriptional regulator
RIP: RNA immunoprecipitation
DMS: Differentially m6A-methylated sites
DMG: Differentially m6A-methylated genes
TCGA: The Cancer Genome Atlas
BIN1: Bridging integrator 1

## References

[1] Sung H, Ferlay J, Siegel RL, Laversanne M, Soerjomataram I, Jemal A, Bray F (2021). Global Cancer statistics 2020: GLOBOCAN estimates of incidence and mortality worldwide for 36 cancers in 185 countries. CA Cancer J Clin.

[2] Meyer KD, Saletore Y, Zumbo P, Elemento O, Mason CE, Jaffrey SR (2012). Comprehensive analysis of mRNA methylation reveals enrichment in 3′ UTRs and near stop codons. Cell.

[3] Zhao BS, Roundtree IA, He C (2017). Post-transcriptional gene regulation by mRNA modifications. Nat Rev Mol Cell Biol.

[4] Li T, Hu PS, Zuo Z, Lin JF, Li X, Wu QN, Chen ZH, Zeng ZL, Wang F, Zheng J (2019). METTL3 facilitates tumor progression via an m(6)A-IGF2BP2-dependent mechanism in colorectal carcinoma. Mol Cancer.

[5] Chen X, Xu M, Xu X, Zeng K, Liu X, Pan B, Li C, Sun L, Qin J, Xu T (2020). METTL14-mediated N6-methyladenosine modification of SOX4 mRNA inhibits tumor metastasis in colorectal cancer. Mol Cancer.

[6] Selberg S, Blokhina D, Aatonen M, Koivisto P, Siltanen A, Mervaala E, Kankuri E, Karelson M (2019). Discovery of small molecules that activate RNA methylation through cooperative binding to the METTL3-14-WTAP complex active site. Cell Rep.

[7] Wang X, Tian L, Li Y, Wang J, Yan B, Yang L, Li Q, Zhao R, Liu M, Wang P (2021). RBM15 facilitates laryngeal squamous cell carcinoma progression by regulating TMBIM6 stability through IGF2BP3 dependent. J Exp Clin Cancer Res.

[8] Barros-Silva D, Lobo J, Guimaraes-Teixeira C, Carneiro I, Oliveira J, Martens-Uzunova ES, Henrique R, Jeronimo C (2020). VIRMA-dependent N6-Methyladenosine modifications regulate the expression of long non-coding RNAs CCAT1 and CCAT2 in prostate Cancer. Cancers (Basel).

[9] Li Z, Weng H, Su R, Weng X, Zuo Z, Li C, Huang H, Nachtergaele S, Dong L, Hu C (2017). FTO plays an oncogenic role in acute myeloid leukemia as a N(6)-Methyladenosine RNA Demethylase. Cancer Cell.

[10] Zhang S, Zhao BS, Zhou A, Lin K, Zheng S, Lu Z, Chen Y, Sulman EP, Xie K, Bogler O (2017). M(6) a Demethylase ALKBH5 maintains Tumorigenicity of Glioblastoma stem-like cells by sustaining FOXM1 expression and cell proliferation program. Cancer Cell.

[11] Huang H, Weng H, Sun W, Qin X, Shi H, Wu H, Zhao BS, Mesquita A, Liu C, Yuan CL (2018). Recognition of RNA N(6)-methyladenosine by IGF2BP proteins enhances mRNA stability and translation. Nat Cell Biol.

[12] Zhou D, Tang W, Xu Y, Xu Y, Xu B, Fu S, Wang Y, Chen F, Chen Y, Han Y (2021). METTL3/YTHDF2 m6A axis accelerates colorectal carcinogenesis through epigenetically suppressing YPEL5. Mol Oncol.

[13] Ma L, Zhang X, Yu K, Xu X, Chen T, Shi Y, Wang Y, Qiu S, Guo S, Cui J (2021). Targeting SLC3A2 subunit of system XC(−) is essential for m(6) a reader YTHDC2 to be an endogenous ferroptosis inducer in lung adenocarcinoma. Free Radic Biol Med.

[14] Chen M, Wei L, Law CT, Tsang FH, Shen J, Cheng CL, Tsang LH, Ho DW, Chiu DK, Lee JM (2018). RNA N6-methyladenosine methyltransferase-like 3 promotes liver cancer progression through YTHDF2-dependent posttranscriptional silencing of SOCS2. Hepatology.

[15] Han J, Wang JZ, Yang X, Yu H, Zhou R, Lu HC, Yuan WB, Lu JC, Zhou ZJ, Lu Q (2019). METTL3 promote tumor proliferation of bladder cancer by accelerating pri-miR221/222 maturation in m6A-dependent manner. Mol Cancer.

[16] Cheng M, Sheng L, Gao Q, Xiong Q, Zhang H, Wu M, Liang Y, Zhu F, Zhang Y, Zhang X (2019). The m(6) a methyltransferase METTL3 promotes bladder cancer progression via AFF4/NF-kappaB/MYC signaling network. Oncogene.

[17] Wang Q, Chen C, Ding Q, Zhao Y, Wang Z, Chen J, Jiang Z, Zhang Y, Xu G, Zhang J (2020). METTL3-mediated m(6) a modification of HDGF mRNA promotes gastric cancer progression and has prognostic significance. Gut.

[18] Yue B, Song C, Yang L, Cui R, Cheng X, Zhang Z, Zhao G (2019). METTL3-mediated N6-methyladenosine modification is critical for epithelial-mesenchymal transition and metastasis of gastric cancer. Mol Cancer.

[19] Wang Q, Guo X, Li L, Gao Z, Su X, Ji M, Liu J (2020). N(6)-methyladenosine METTL3 promotes cervical cancer tumorigenesis and Warburg effect through YTHDF1/HK2 modification. Cell Death Dis.

[20] Du L, Li Y, Kang M, Feng M, Ren Y, Dai H, Wang Y, Wang Y, Tang B (2021). USP48 is upregulated by Mettl14 to attenuate hepatocellular carcinoma via regulating SIRT6 stabilization. Cancer Res.

[21] Chen S, Yang C, Wang ZW, Hu JF, Pan JJ, Liao CY, Zhang JQ, Chen JZ, Huang Y, Huang L (2021). CLK1/SRSF5 pathway induces aberrant exon skipping of METTL14 and Cyclin L2 and promotes growth and metastasis of pancreatic cancer. J Hematol Oncol.

[22] Wang F, Liao Y, Zhang M, Zhu Y, Wang W, Cai H, Liang J, Song F, Hou C, Huang S (2021). N6-methyladenosine demethyltransferase FTO-mediated autophagy in malignant development of oral squamous cell carcinoma. Oncogene.

[23] Bian X, Shi D, Xing K, Zhou H, Lu L, Yu D, Wu W (2021). AMD1 upregulates hepatocellular carcinoma cells stemness by FTO mediated mRNA demethylation. Clin Transl Med.

[24] Tao L, Mu X, Chen H, Jin D, Zhang R, Zhao Y, Fan J, Cao M, Zhou Z (2021). FTO modifies the m6A level of MALAT and promotes bladder cancer progression. Clin Transl Med.

[25] Su R, Dong L, Li C, Nachtergaele S, Wunderlich M, Qing Y, Deng X, Wang Y, Weng X, Hu C (2018). R-2HG exhibits anti-tumor activity by targeting FTO/m(6)a/MYC/CEBPA signaling. Cell.

[26] Dang Y, Xu J, Yang Y, Li C, Zhang Q, Zhou W, Zhang L, Ji G (2020). Ling-gui-zhu-Gan decoction alleviates hepatic steatosis through SOCS2 modification by N6-methyladenosine. Biomed Pharmacother.

[27] Dang Y, Hu D, Xu J, Li C, Tang Y, Yang Z, Liu Y, Zhou W, Zhang L, Xu H (2020). Comprehensive analysis of 5-hydroxymethylcytosine in zw10 kinetochore protein as a promising biomarker for screening and diagnosis of early colorectal cancer. Clin Transl Med.

[28] Dang Y, Xu J, Zhu M, Zhou W, Zhang L, Ji G (2020). Gan-Jiang-Ling-Zhu decoction alleviates hepatic steatosis in rats by the miR-138-5p/CPT1B axis. Biomed Pharmacother.

[29] Brenner H, Hoffmeister M, Stegmaier C, Brenner G, Altenhofen L, Haug U (2007). Risk of progression of advanced adenomas to colorectal cancer by age and sex: estimates based on 840,149 screening colonoscopies. Gut.

[30] Mao X, Li P, Wang Y, Liang Z, Liu J, Li J, Jiang Y, Bao G, Li L, Zhu B (2017). CRB3 regulates contact inhibition by activating the hippo pathway in mammary epithelial cells. Cell Death Dis.

[31] Fernando RN, Cotter L, Perrin-Tricaud C, Berthelot J, Bartolami S, Pereira JA, Gonzalez S, Suter U, Tricaud N (2016). Optimal myelin elongation relies on YAP activation by axonal growth and inhibition by Crb3/hippo pathway. Nat Commun.

[32] Burgers K, Moore C, Bednash L (2018). Care of the Colorectal Cancer Survivor. Am Fam Physician.

[33] Chen C, Yuan W, Zhou Q, Shao B, Guo Y, Wang W, Yang S, Guo Y, Zhao L, Dang Q (2021). N6-methyladenosine-induced circ1662 promotes metastasis of colorectal cancer by accelerating YAP1 nuclear localization. Theranostics.

[34] Ma JZ, Yang F, Zhou CC, Liu F, Yuan JH, Wang F, Wang TT, Xu QG, Zhou WP, Sun SH (2017). METTL14 suppresses the metastatic potential of hepatocellular carcinoma by modulating N(6) -methyladenosine-dependent primary MicroRNA processing. Hepatology.

[35] Chen H, Gao S, Liu W, Wong CC, Wu J, Wu J, Liu D, Gou H, Kang W, Zhai J (2021). RNA N(6)-Methyladenosine methyltransferase METTL3 facilitates colorectal Cancer by activating the m(6)A-GLUT1-mTORC1 Axis and is a therapeutic target. Gastroenterology.

[36] Chang YZ, Chai RC, Pang B, Chang X, An SY, Zhang KN, Jiang T, Wang YZ (2021). METTL3 enhances the stability of MALAT1 with the assistance of HuR via m6A modification and activates NF-kappaB to promote the malignant progression of IDH-wildtype glioma. Cancer Lett.

[37] Huang S, Luo S, Gong C, Liang L, Xiao Y, Li M, He J (2021). MTTL3 upregulates microRNA-1246 to promote occurrence and progression of NSCLC via targeting paternally expressed gene 3. Mol Ther Nucleic Acids.

[38] Huo FC, Zhu ZM, Zhu WT, Du QY, Liang J, Mou J (2021). METTL3-mediated m(6) a methylation of SPHK2 promotes gastric cancer progression by targeting KLF2. Oncogene.

[39] Li H, Zhang N, Jiao X, Wang C, Sun W, He Y, Ren G, Huang S, Li M, Chang Y (2021). Downregulation of microRNA-6125 promotes colorectal cancer growth through YTHDF2-dependent recognition of N6-methyladenosine-modified GSK3beta. Clin Transl Med.

[40] Karp CM, Tan TT, Mathew R, Nelson D, Mukherjee C, Degenhardt K, Karantza-Wadsworth V, White E (2008). Role of the polarity determinant crumbs in suppressing mammalian epithelial tumor progression. Cancer Res.

[41] Iioka H, Saito K, Kondo E (2019). Crumbs3 regulates the expression of glycosphingolipids on the plasma membrane to promote colon cancer cell migration. Biochem Biophys Res Commun.

[42] Li P, Feng C, Chen H, Jiang Y, Cao F, Liu J, Liu P (2018). Elevated CRB3 expression suppresses breast cancer stemness by inhibiting beta-catenin signalling to restore tamoxifen sensitivity. J Cell Mol Med.

[43] Szymaniak AD, Mahoney JE, Cardoso WV, Varelas X (2015). Crumbs3-mediated polarity directs airway epithelial cell fate through the hippo pathway effector yap. Dev Cell.

[44] Jin L, Chen Y, Cheng D, He Z, Shi X, Du B, Xi X, Gao Y, Guo Y (2021). YAP inhibits autophagy and promotes progression of colorectal cancer via upregulating Bcl-2 expression. Cell Death Dis.

[45] Shen H, Huang C, Wu J, Li J, Hu T, Wang Z, Zhang H, Shao Y, Fu Z (2021). SCRIB promotes proliferation and metastasis by targeting hippo/YAP Signalling in colorectal Cancer. Front Cell Dev Biol.

[46] Tian C, Lang T, Qiu J, Han K, Zhou L, Min D, Zhang Z, Qi D (2020). SKP1 promotes YAP-mediated colorectal cancer stemness via suppressing RASSF1. Cancer Cell Int.

[47] Sun Z, Zhang Q, Yuan W, Li X, Chen C, Guo Y, Shao B, Dang Q, Zhou Q, Wang Q (2020). MiR-103a-3p promotes tumour glycolysis in colorectal cancer via hippo/YAP1/HIF1A axis. J Exp Clin Cancer Res.

[48] Yankova E, Blackaby W, Albertella M, Rak J, De Braekeleer E, Tsagkogeorga G, Pilka ES, Aspris D, Leggate D, Hendrick AG (2021). Small molecule inhibition of METTL3 as a strategy against myeloid leukaemia. Nature.

