# Supplementary material for: METTL3 promotes colorectal carcinoma progression by regulating the m6A–CRB3–Hippo axis
Source: J Exp Clin Cancer Res. 2022 Jan 10;41:19. doi: 10.1186/s13046-021-02227-8 (PMC8744223; doi:10.1186/s13046-021-02227-8)
Supplement: Supplementary file 4 — Additional file 4: Table S1. Primer sequences of mRNAs in real-time PCR experiments. Table S2. Correlation between N6-methyladnosine (m6A) level and multiple clinical characteristics. Table S3. Cox regression analyses of m6A level in patients with CRC. Table S4. Correlation between methyltransferase-like 3 (METTL3) expression and multiple clinical characteristics. Table S5. Cox regression analyses of METTL3 expression in patients with CRC. Table S6. Primer sequences of MeRIP-qPCR and RIP. [file 13046_2021_2227_MOESM4_ESM.docx]

Table S1. Primers sequences of mRNAs in Real-time PCR experiments.

| RNAs | Primer(5'->3') |
| --- | --- |
| METTL3 | Forward: CAGTTGATTTGTCTTCACCCA  Reverse: CCCTGACAGATGATGAGATGC |
| METTL14 | Forward: GTCTTAGTCTTCCCAGGATTGTTTT  Reverse: AATTGATGAGATTGCAGCACC |
| FTO | Forward: GACCTGTCCACCAGATTTTCA  Reverse: AGCAGAGCAGCATACAACGTA |
| ALKBH5 | Forward: ACTGAGCACAGTCACGCTTCC  Reverse: GCCGTCATCAACGACTACCAG |
| WTAP | Forward: TACACTTTCATACCCCGCACT  Reverse: CTGACAAACGGACCAAGTAAT |
| CRB3 | Forward: GGTCTTCTCCCTCTTGGCT  Reverse: CCACCTGCTCCTCGCTA |
| BIN1 | Forward: CAGGGATGAGGCAAACAAG  Reverse: TGATGTCGGGGAACTGG |
| SLC47A2 | Forward: ACGCCCTTTCAGCTCCTAC  Reverse: GAGCCCCACCATCAGTGT |
| HNRNPUL1 | Forward: AACTGCGACCTCCACTTCA  Reverse: AGCATACACGGCCCCTT |
| RIPOR2 | Forward: TCCAGGACTTGCAGACCCA  Reverse: CGACTTCCGAGGACAGGCT |
| RASGRP4 | Forward: TGGGCAAAAAGCGCAAAGTG  Reverse: GGAAGGACCGGAACTCCAG |
| VEGFA | Forward: GATGTCCACCAGGGTCTC  Reverse: TTGCTGCTCTACCTCCAC |
| YTHDF2 | Forward: AGCCCCACTTCCTACCAGATG  Reverse: TGAGAACTGTTATTTCCCCATGC |
| β-actin | Forward: GAAGAGCTACGAGCTGCCTGA  Reverse: CAGACAGCACTGTGTTGGCG |

Table S2 Correlation between m6A level and different clinical characteristics

|  | m6A | |  |
| --- | --- | --- | --- |
| Characteristics | Low Expression (%) (N=96) | High Expression (%) (N=105) | *p* value |
| **Sex** |  |  | 0.42 |
| Female | 47 | 46 |  |
| Male | 49 | 59 |  |
| **Age** | 68±11 | 68±10 | 0.686 |
| **Invasion depth** |  |  | 0.827 |
| T1, T2 | 4 | 6 |  |
| T3, T4 | 92 | 99 |  |
| **Lymph node metastasis** |  |  | 0.086 |
| N0 | 60 | 53 |  |
| N1, N2 | 36 | 52 |  |
| **Pathologic stage** |  |  |  |
| Ⅰ-Ⅱ | 69 | 85 | 0.129 |
| Ⅲ-Ⅳ | 27 | 20 |  |
| **Clinic stage** |  |  |  |
| Ⅰ-Ⅱ | 59 | 53 | 0.117 |
| Ⅲ-Ⅳ | 37 | 52 |  |

Table S3 Cox regression analyses of m6A level in CRC patients

| **Variate** | Univariate Cox regression | | Multivariate Cox regression | |
| --- | --- | --- | --- | --- |
|  | **HR(95%CI)** | ***P*** | **HR(95%CI)** | ***P*** |
|  |  |  |  |  |
| Sex (Male) | 1.114(0.755, 1.643) | 0.586 | 1.251(0.838, 1.869) | 0.274 |
| Age | 1.020(1.000, 1.040) | 0.045 | 1.028(1.008, 1.049) | 0.007 |
| Pathologic stage |  |  |  |  |
| Ⅰ | 1(REF) |  | 1(REF) |  |
| Ⅱ | 1275.688(0.00, 2.569e^41^) | 0.87 | 1101.22(0.00-2.048e^48^) | 0.895 |
| Ⅲ | 2257.877(0.00, 3.587e^40^) | 0.86 | 2252.709(0.00, 4191e^48^) | 0.885 |
| Ⅳ | 15801.866(0.00, 2.026e^40^) | 0.825 | 8966.354(0.00-1.702e^49^) | 0.864 |
| TNM stage |  |  |  |  |
| Ⅰ | 1(REF) |  | 1(REF) |  |
| Ⅱ | 2.501(0.606, 10.319) | 0.205 | 2.965(0.711, 12.631) | 0.136 |
| Ⅲ | 4.684(1.140, 19.243) | 0.032 | 5.126(1221, 21.520) | 0.026 |
| Ⅳ | 12.951(2.597, 64.573) | 0.002 | 18.589(3.659, 94.424) | <0.001 |
| m6A | 1.998(1.332, 2.995) | 0.001 | 1.923(1.270, 2.912) | 0.002 |

Table S4 Correlation between METTL3 level and different clinical characteristics

|  | METTL3 | |  |
| --- | --- | --- | --- |
| Clinicopathological parameter | Low Expression (%) (N=11) | High Expression (%) (N=89) | *p* value |
| **Sex** |  |  | 0.718 |
| Female | 4 | 38 |  |
| Male | 7 | 51 |  |
| **Age** | 66±13 | 68±11 | 0.686 |
| **Invasion depth** |  |  | 0.536 |
| T1, T2 | 0 | 3 |  |
| T3, T4 | 11 | 86 |  |
| **Lymph node metastasis** |  |  | 0.413 |
| N0 | 7 | 45 |  |
| N1, N2 | 4 | 44 |  |
| **Pathologic stage** |  |  |  |
| Ⅰ-Ⅱ | 6 | 64 | 0.236 |
| Ⅲ-Ⅳ | 5 | 25 |  |
| **Clinic stage** |  |  |  |
| Ⅰ-Ⅱ | 7 | 44 | 0.374 |
| Ⅲ-Ⅳ | 4 | 45 |  |

Table S5 Cox regression analyses of METTL3 expression in CRC patients

| **Variate** | Univariate Cox regression | | Multivariate Cox regression | |
| --- | --- | --- | --- | --- |
|  | **HR(95%CI)** | ***P*** | **HR(95%CI)** | ***P*** |
|  |  |  |  |  |
| Sex (Male) | 1.311(0.743, 2.313) | 0.351 | 1.622(0.877, 3.002) | 0.123 |
| Age | 1.015(0.990, 1.041) | 0.255 | 1.016(0.989, 1.044) | 0.258 |
| Pathologic stage |  |  |  |  |
| Ⅰ | / | / | / | / |
| Ⅱ | 1(REF) |  | 1(REF) |  |
| Ⅲ | 1.350(0.756, 2.411) | 0.31 | 0.498(0.261, 0.923) | 0.027 |
| Ⅳ | / | / | / | / |
| TNM stage |  |  |  |  |
| Ⅰ | 1(REF) |  | 1(REF) |  |
| Ⅱ | 1.927(0.259, 14.353) | 0.527 | 1.773(0.232, 13.531) | 0.581 |
| Ⅲ | 2.798(0.379, 20.655) | 0.313 | 2.602(0.338, 20.046) | 0.359 |
| Ⅳ | 11.643(1.340, 101.188) | 0.026 | 12.131(1.387, 106.077) | 0.024 |
| METTL3 | 8.658(1.196, 62.705) | 0.033 | 10.298(1.390, 76.277) | 0.022 |

Table S6. Primers sequences of MeRIP-qPCR and RIP

| RNAs | Primer(5'->3') |
| --- | --- |
| CRB3 | Forward: CATGTTGCCTCTGCTTGG  Reverse: CCTGCACCGTCTCCTTG |
